# A dataset on microbiome alterations in *Drosophila melanogaster* infected by entomopathogenic nematodes

**DOI:** 10.1016/j.dib.2026.112794

**Published:** 2026-04-27

**Authors:** Sreeradha Mallick, Christina Pavloudi, George Joseph Chakkalakkal, Vladimir Lažetić, Jimmy Saw, Ioannis Eleftherianos

**Affiliations:** a School of Biological Sciences, Queen’s University Belfast, Belfast, UK; b Department of Biological Sciences, The George Washington University, Washington DC, USA; c European Marine Biological Resource Centre- European Research Infrastructure Consortium (EMBRC-ERIC), Paris, France

**Keywords:** *Heterorhabditis*, Infection, Pathogenicity, Host response, Immunity, Symbiosis

## Abstract

The fruit fly *Drosophila melanogaster* is an excellent model for dissecting the molecular processes that regulate host-microbe interactions and the role of the microbiome in host homeostasis. More recently, the fly has also been used as a model for understanding entomopathogenic nematode infection and host response against these parasites. To gain insights into the effect of entomopathogenic nematode infection on the insect microbiome, *D. melanogaster* larvae were exposed to *Heterorhabditis bacteriophora* containing their symbiotic bacteria *Photorhabdus luminescens* (symbiotic worms) and nematodes lacking their bacterial symbionts (axenic worms). Microbiome changes were examined through 16S rRNA sequencing. Data were collected at 24- and 48-hours following infection of *D. melanogaster* larvae with either type of nematode. The complete set of raw sequencing data generated in this study has been deposited in the European Nucleotide Archive under accession number PRJEB85826.

Specifications Table

**Table:** 

Subject	Biology
Specific subject area	Microbiology, Insect Biology, Microbiome
Type of data	Microbiome data, Analyzed, Figures
Data collection	*Drosophila melanogaster* third instar larvae were individually infected with 100 symbiotic or axenic *Heterorhabditis bacteriophora* infective juveniles. Three biological replicates were prepared for each experimental treatment. *Drosophila melanogaster* nematode-infected and uninfected control larvae were collected at 24- and 48-hour time points. DNA was extracted from all larvae, amplicon libraries were prepared, normalized and sequenced.
Data source location	The George Washington University, Washington, DC
Data accessibility	Raw and analyzed sequence files were deposited to the European Nucleotide Archive (ENA) with the accession number PRJEB85826. (Available at http://www.ebi.ac.uk/ena/data/view/PRJEB85826).
Related research article	S. Mallick, C. Pavloudi, J. Saw, I. Eleftherianos (2025), *Heterorhabditis bacteriophora* symbiotic and axenic nematodes modify the *Drosophila melanogaster* larval microbiome, Front. Microbiol. 16:1598221. doi: 10.3389/fmicb.2025.1598221 [1].

## 1 Value of the Data

• The presented data provide a comprehensive overview of changes in the structure and composition of the *D. melanogaster* larval microbiome during infection with potent entomopathogenic nematodes.
• The dataset and accompanying analysis will be useful to insect biologists and microbiologists who explore the precise role of the microbiome in insect homeostasis.
• Results in these datasets can be further mined to identify specific bacterial species of the microbiome that regulate physiological processes in *D. melanogaster* and other insects. For instance, the abundance of certain bacterial species may provide clues about their participation in host defense against nematode parasites and other pathogens.

## 2 Background

The fruit fly *Drosophila melanogaster* is a valuable model for exploring host-pathogen interactions. Previous work has used the *D. melanogaster* model to decipher the molecular basis of pathogen infection and host innate immunity [2]. More recently, significant progress has been made in elucidating the function of effector molecules released by parasitic nematodes and their effect on the fly immune response [3]. *Heterorhabditis bacteriophora*, an entomopathogenic nematode (EPN) which carries the symbiotic bacterium *Photorhabdus luminescens*, infects the fly through its infective juvenile (IJ) stage, providing a system to investigate anti-nematode defense in insects [4]. The *D. melanogaster* larval microbiome plays a critical role in maintaining host physiology; however, its composition can be reshaped by exposure to pathogens, such as viruses, bacteria, and fungi [[5], [6], [7]]. Here, we have explored changes in the composition of the *D. melanogaster* larval microbiome during infection with *H. bacteriophora* nematodes containing or lacking their associated symbiotic bacterium *P. luminescens* (referred to as symbiotic and axenic nematodes, respectively). To address this, we conducted high-throughput sequencing and amplicon-based analysis to profile microbial communities in both nematode-infected and uninfected larvae. Samples were obtained from *D. melanogaster* larvae at 24 and 48 h post-infection with symbiotic or axenic nematodes.

## 3 Data Description

**Fig. 1:**
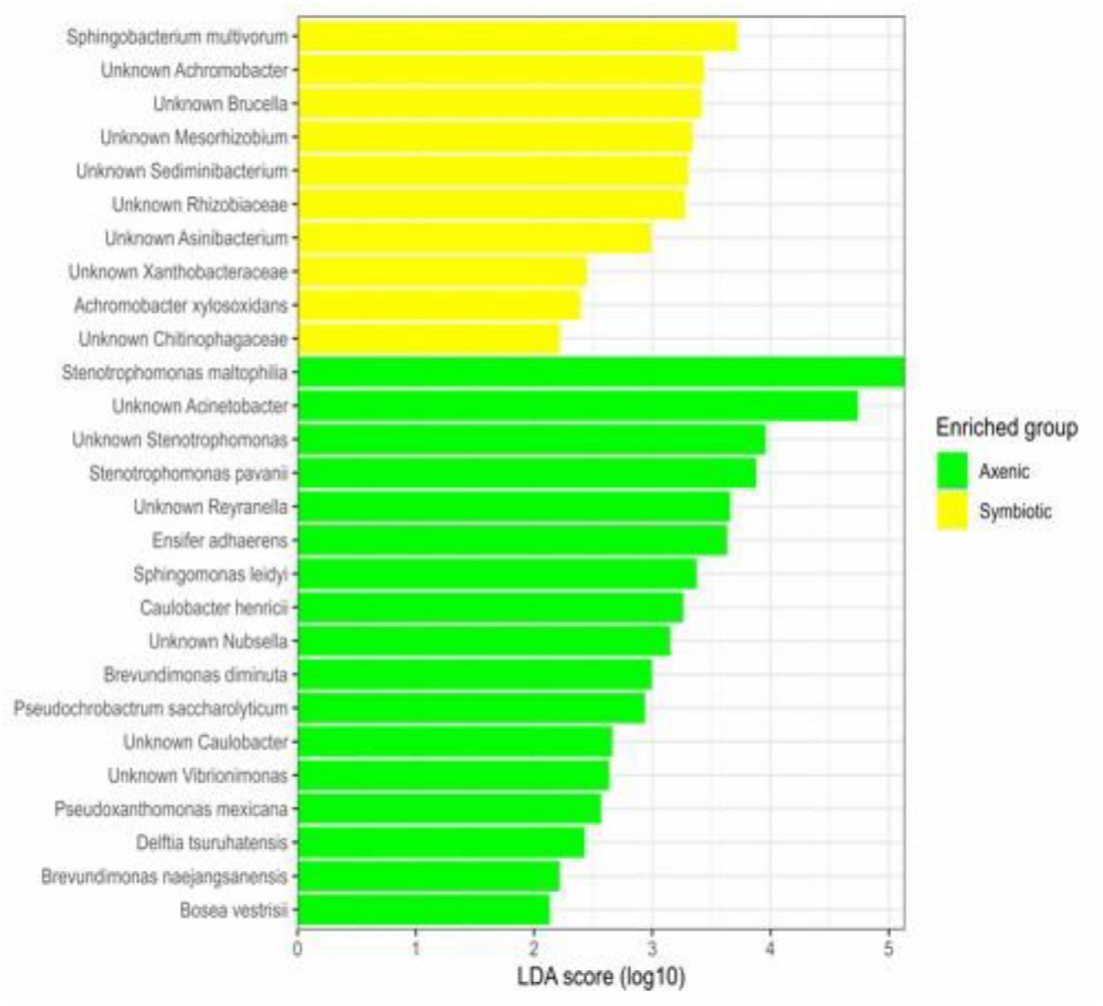
Differentially enriched bacterial species observed in the *Drosophila melanogaster* larval microbiome associated with *Heterorhabditis bacteriophora* infection status. The linear discriminant analysis effect size (LEfSe) bar plot illustrates those bacterial taxa that are significantly concentrated in larvae infected with either axenic or symbiotic nematodes. The data correspond to the microbiome of 100 pooled *D. melanogaster* larvae per replicate, across three independent biological replicates (n = 3), sampled at two distinct time points (24 and 48 h).

Linear discriminant analysis (LDA) revealed bacterial taxa that were differentially abundant between the two groups of *D. melanogaster* larvae infected with the EPNs: one group was exposed to symbiotic *H. bacteriophora*, and the other group was treated with axenic nematodes. The analysis involved both treatment types (axenic vs. symbiotic nematode-infected larvae) and sampling time (24 and 48 h) as variables. Interestingly, *Stenotrophomonas maltophilia* was predominantly enriched in larvae infected with axenic nematodes, whereas *Sphingobacterium multivorum* showed higher abundance in larvae infected with symbiotic nematodes (Fig. 1).

**Fig. 2:**
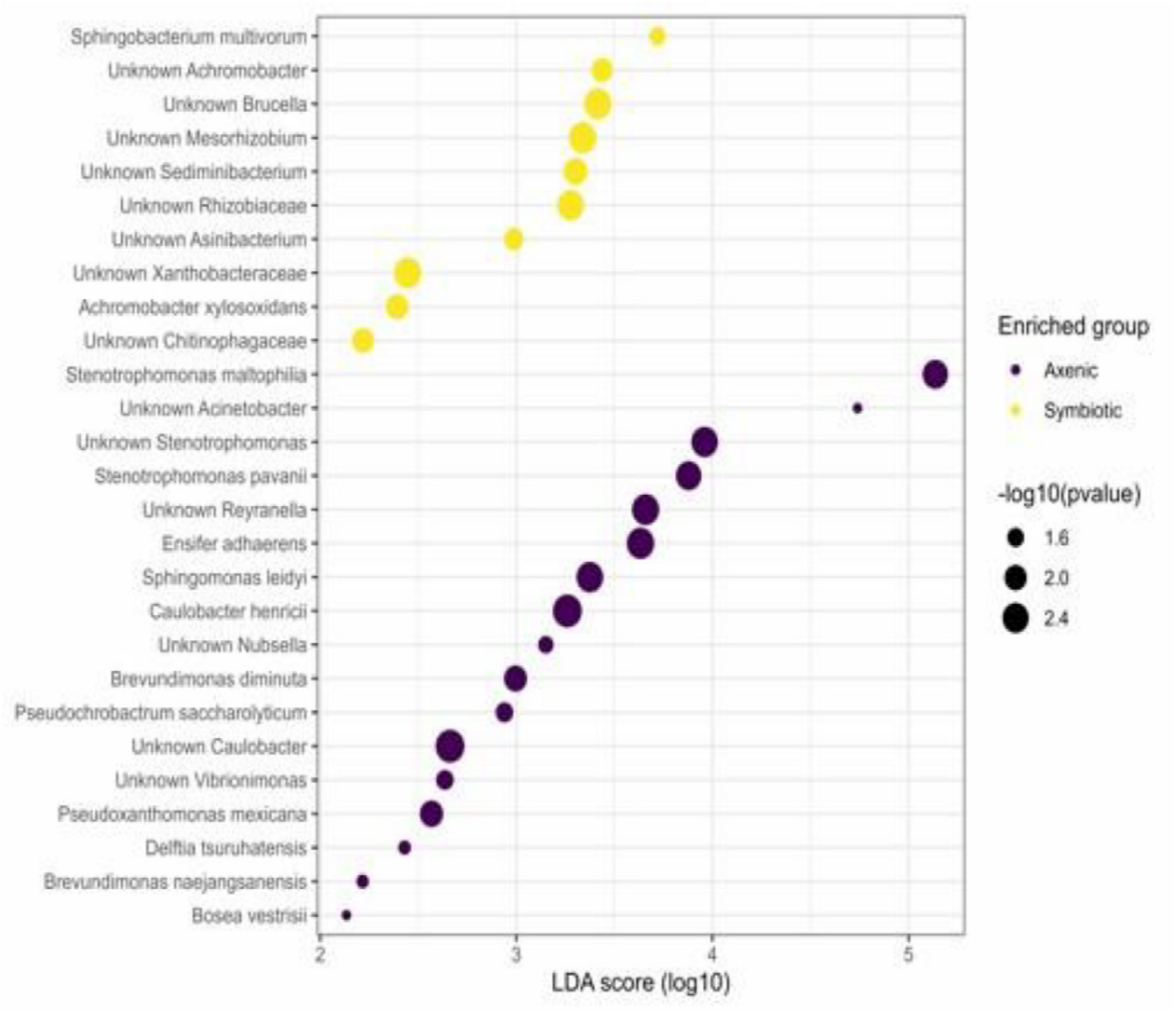
Differential bacterial community composition in *Heterorhabditis bacteriophora*-infected *Drosophila melanogaster* larvae. The LEfSe dot plot depicts the differences in bacterial species abundance between *D. melanogaster* larvae infected with symbiotic versus axenic nematodes. The Linear Discriminant Analysis (LDA) points out bacterial taxa that are distinctly concentrated and differentially abundant between the two infection treatment conditions. The data represent the microbiome of 100 pooled *D. melanogaster* larvae per replicate, across three independent biological replicates (n = 3), collected at two different time points (24 and 48 h).

The accompanying LEfSe dot plot further visualizes the differences in bacterial abundance between the two infection treatments. Remarkably, *Stenotrophomonas maltophilia* was identified as a key biomarker, showing particularly greater relative abundance in larvae infected with axenic *H. bacteriophora* compared to those infected with symbiotic *H. bacteriophora* (Fig. 2).

**Fig. 3:**
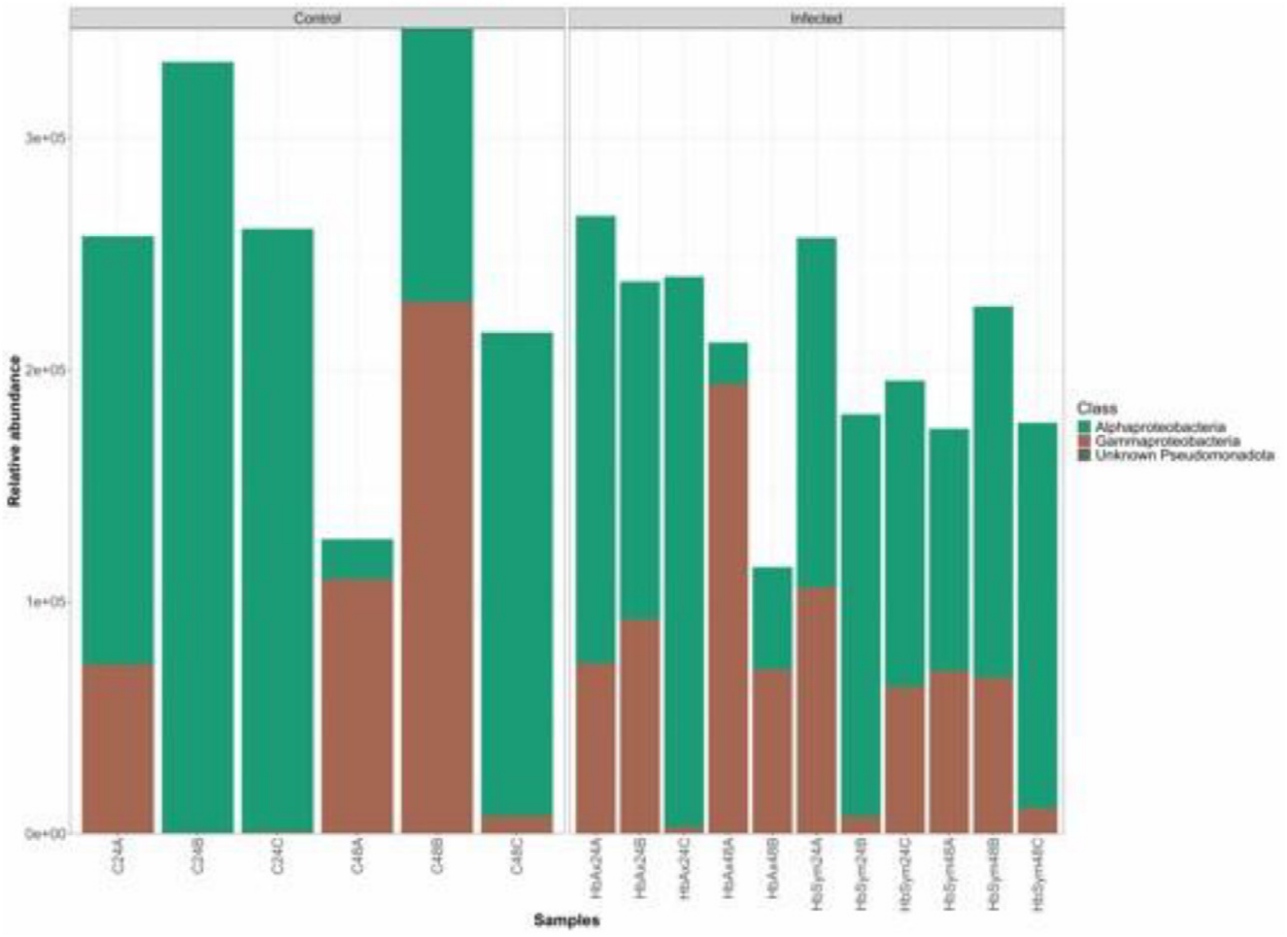
Distribution of bacterial taxa within the *Drosophila melanogaster* larval microbiome. The bar graph illustrates the proportional representation of multiple classes of *Pseudomonadota* across the three treatment conditions: larvae exposed to axenic *Heterorhabditis bacteriophora* (Infected: HbAx; 24 or 48 h; A, B, C indicates the replicates), larvae exposed to symbiotic nematodes (Infected: HbSym; 24 or 48 h; A, B, C indicates the replicates), and the uninfected larvae (Control). The data present the microbiome of 100 pooled *D. melanogaster* larvae per replicate, across three independent biological replicates (n = 3), sampled at two time points, 24 and 48 h post-treatment.

The bar chart highlights the relative abundance of major bacterial classes across three experimental groups - axenic *H. bacteriophora*-infected larvae, symbiotic *H. bacteriophora*-infected larvae, and uninfected controls, at 24- and 48-hours post-infection. Similar distribution patterns of *Pseudomonadota* classes were observed among both the infected groups and the uninfected controls at both time-points (Fig. 3).

**Fig. 4:**
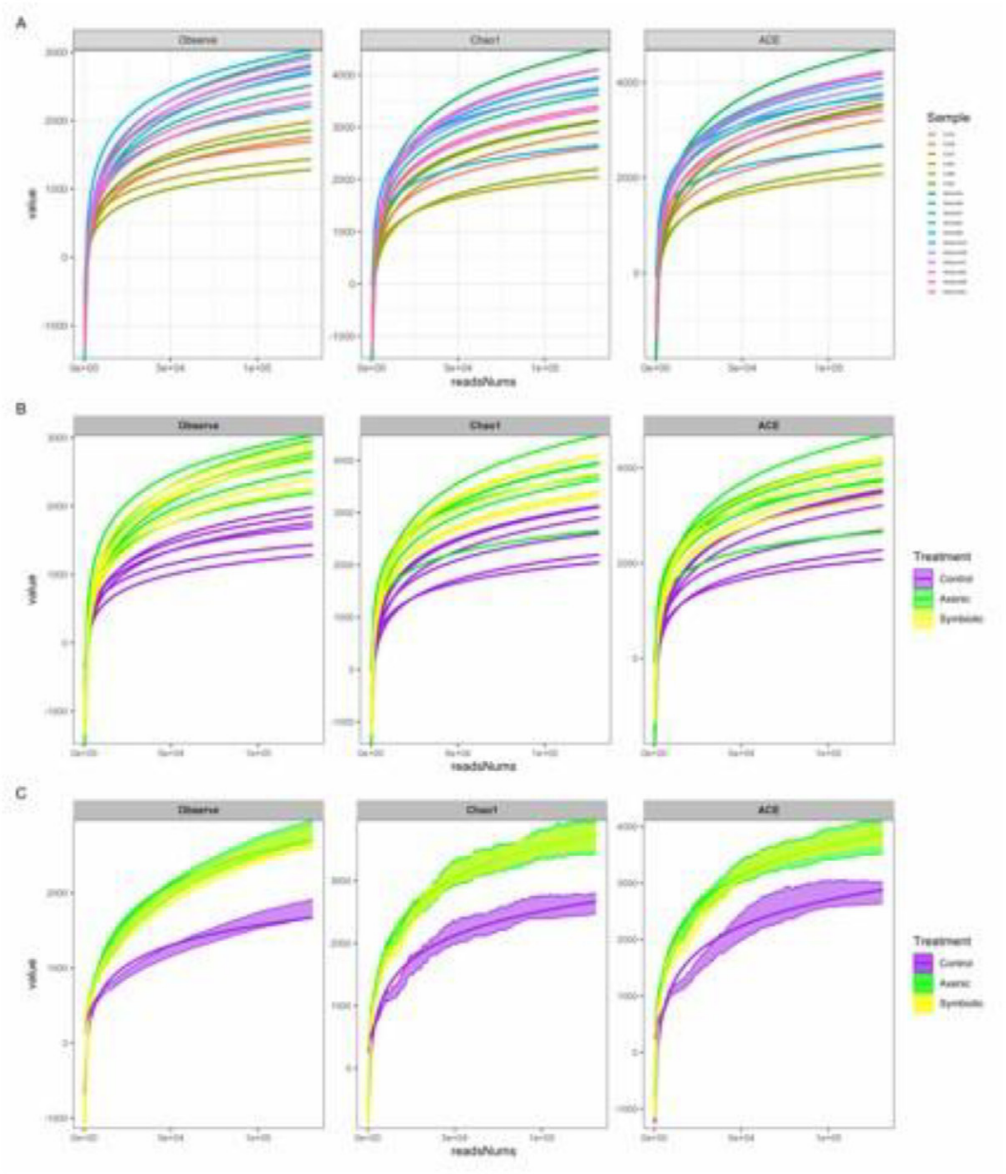
Rarefaction analysis contrasting uninfected control and *Heterorhabditis bacteriophora*-infected *Drosophila melanogaster* larval groups. [A] Sample-level rarefaction plots showing alpha diversity metrics (Observed ASVs, Chao1, and ACE) on the y-axis against sequencing depth on the x-axis. [B] Rarefaction profiles for individual samples, distinguished by color according to the treatment category. [C] Treatment-level rarefaction curves elucidating the mean diversity estimates with corresponding standard error. The data correspond to the microbiome of 100 pooled *D. melanogaster* larvae per replicate, across three independent biological replicates (n = 3), sampled at two distinct time points (24 and 48 h).

The horizontal axis corresponds to the sequencing depth, whereas the vertical axis represents the alpha diversity metrics in the rarefaction curves. Panel (A) illustrates the rarefaction curves for each sample, while panel (B) highlights the curves according to the sample group, enabling easier visual comparison. Panel (C) represents the group-level rarefaction curves along with the standard error of the mean, offering insight into within-group variability. These findings indicate that the sequencing effort is adequate to allow robust and reliable comparisons of alpha diversity among samples in the subsequent analyses (Fig. 4).

**Fig. 5:**
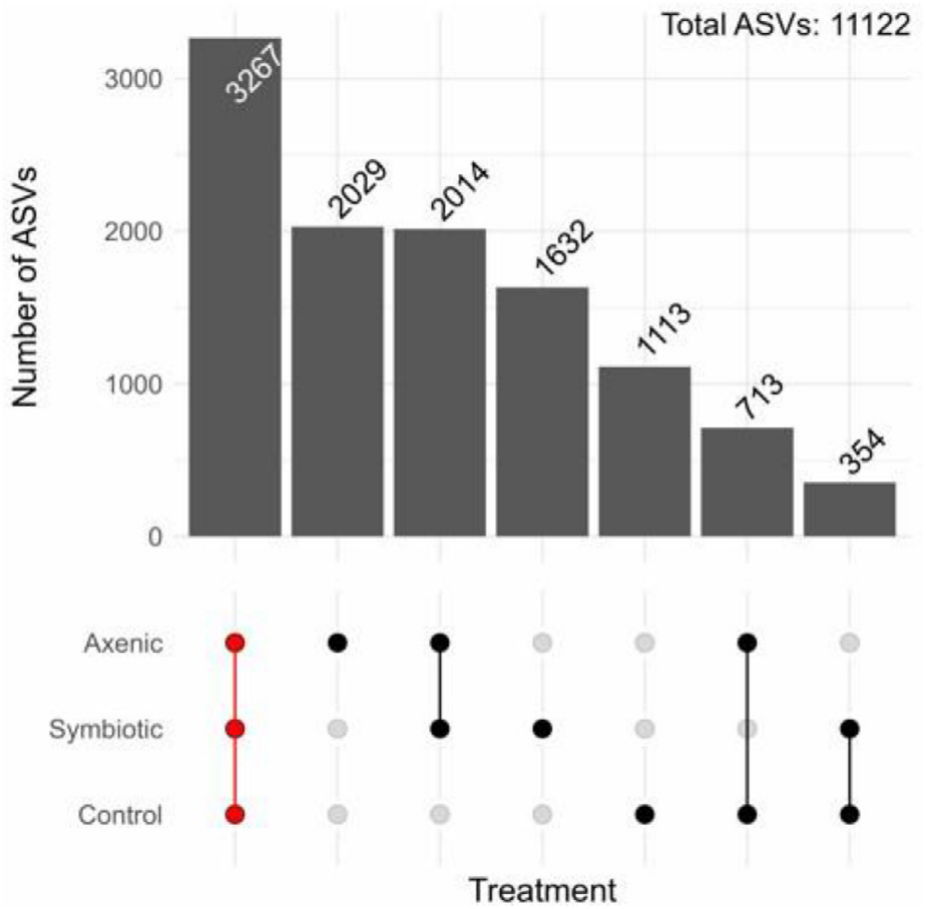
Composition and sharing patterns of the Amplicon Sequence Variants (ASVs) detected in the *Drosophila melanogaster* larvae following infection with either axenic or symbiotic *Heterorhabditis bacteriophora*, compared to the uninfected controls. The UpSet plot maps the number of ASVs unique to each treatment group as well as those which are common across the infected and the uninfected larvae, highlighting the similarities and distinctions in the microbial community structure among the different experimental conditions. ASVs were derived from the microbiome of 100 pooled *D. melanogaster* larvae per replicate, across three independent biological replicates (n = 3), collected at two distinct time points (24 and 48 h).

The UpSet plot depicts the distribution of unique and overlapping Amplicon Sequence Variants (ASVs) across the three experimental conditions: *D. melanogaster* larvae exposed to axenic *H. bacteriophora*, larvae exposed to symbiotic nematodes, and the uninfected control larvae. The number of ASVs that was observed to be common to all treatment groups was 3267, which represents the core *D. melanogaster* larval microbiome. Furthermore, larvae infected with axenic *H. bacteriophora* harbor the largest number of distinct ASVs, totalling 2029 (Fig. 5).

## 4 Experimental Design, Materials and Methods

### 4.1 Fly lines

The *D. melanogaster w^1118^* line was kept on standard fly food (Fly Food B, LabExpress, Ann Arbor, MI, USA), which was supplemented with yeast (Carolina Biological Supply, Burlington, NC, USA). Larvae at the third-instar were selected for the nematode infection assays. To amplify the *w^1118^* stock, flies were maintained at 25 °C and a 12-hour light:dark photoperiod.

### 4.2 Entomopathogenic nematodes

The *H. bacteriophora* TT01 nematode stock was kept and amplified through the third larval stage of the greater wax moth, *Galleria mellonella*. Infections were conducted using the nematode infective juvenile stage in 6 cm Petri dishes (Avantor, Radnor, PA, USA). The infected *G. mellonella* larvae were kept for seven days at 25 °C and a 12-hour light:dark photoperiod before they were moved to water traps following a previously described procedure [8]. The new generation of *H. bacteriophora* infective juveniles appeared two weeks later and the nematodes were stored in 50 mL tissue culture flasks (Avantor, Radnor, PA, USA) and maintained at 28 °C until used in infection experiments.

### 4.3 Infection of fly larvae with entomopathogenic nematodes

*Drosophila melanogaster* larvae of the *w^1118^* line were infected with either *H. bacteriophora* symbiotic nematodes (containing the symbiotic *P. luminescens* bacteria) or *H. bacteriophora* axenic nematodes (lacking the *P. luminescens* bacteria). Both types of nematodes kill *D. melanogaster w^1118^* larvae at a similar rate (our unpublished data). The infections were carried out in 96-well microplates (ThermoFisher Scientific, Waltham, MA, USA) containing 100 µL of 1.25% agarose gel (Fisher Scientific, Waltham, MA, USA). *Drosophila melanogaster* larvae were transferred to the microplate wells using a paint brush. The nematode infection setup involved exposure of a single *D. melanogaster* third instar larva to 100 *H bacteriophora* infective juveniles, which were pipetted in a 10 µL of sterile water suspension. *Drosophila melanogaster* larvae treated with sterile water only acted as uninfected controls. Nematode-infected and uninfected larvae were kept in an incubator set at 25 °C and a 12-hour light:dark cycle. The nematode infection experiments were performed three times, and each experimental condition included three replicates, with each replicate consisting of 20 *D melanogaster* larvae.

### 4.4 DNA isolation

Live *D. melanogaster* larvae were sampled at 24- and 48-hours following infection with either symbiotic or axenic *H. bacteriophora* nematodes or treatment with sterile water (uninfected controls). Axenic *H. bacteriophora* were generated through propagation in *G. mellonella* larvae infected with the *Photorhabdus temperata* mutant strain RET16, which supports the growth of these nematodes without colonizing them, as described before [9]. The axenic status of *H. bacteriophora* was confirmed by homogenizing the nematodes, spreading the lysate on selective media, and observing the absence of *P. luminescens* bacterial colony growth on the plates [10]. All *D. melanogaster* larvae were stored in 1.5 mL plastic vials (Eppendorf, Framingham, MA, USA) and kept at −80 °C. Samples for DNA extraction were isolated from 100 pooled nematode-infected or uninfected larvae per experimental replicate following the method described in the commercial Qiagen DNA extraction kit (Qiagen, Germantown, MD, USA). The quantity and quality of DNA samples were evaluated using the Qubit 4 Fluorometer (ThermoFisher Scientific, Waltham, MA, USA). Concentration of DNA samples ranged from 20 ng/µL to 100 ng/µL, and their purity was between 1.45 and 1.91.

### 4.5 Library construction and sequencing

For the construction of amplicon libraries, the Zymo Research’s Quick-16S NGS Library Prep kit (Zymo Research, Irvine, CA, USA) (Primer Set V3-V4) was used together with the 341F and 806R primer pair to create a 16S library. Samples were cleaned up and normalized before sequencing on a P1 600cyc NextSeq2000 Flowcell. Sequencing of 16S rRNA produced 2 × 301 bp paired-end (PE) reads. Library construction and sequencing were performed at SeqCenter (SeqCenter, LLC; Pittsburgh, PA, USA). Adapter sequences were trimmed using the BCL Convert v4.2.4 software. Raw sequences were submitted to the European Nucleotide Archive (ENA) with the accession number PRJEB85826 (see http://www.ebi.ac.uk/ena/data/view/ PRJEB85826) [11].

### 4.6 Analysis of amplicon sequencing data

All analyses were conducted using the R software (version 4.4.2). Filtering and trimming of sequences, amplicon sequence variants (ASVs) inference, sequencing error removal, and chimeric sequence elimination was performed using DADA2. ASVs related to mitochondria and chloroplasts were excluded from further processing. SILVA release 138.2 was used for taxonomic classification of the ASVs [12]. Alpha and beta diversity were determined with the Phyloseq package (version 1.42.0) [13]. More precisely, the Observed Species Chao1 and abundance-based coverage estimator (ACE) indices were used for calculating alpha diversity [14,15]. The microbiome Marker package (version 1.3.2) and Linear Discriminant effect Size analysis (LEfSe) were used to establish significant differences in taxonomic groups among the experimental treatments [16,17]. UpSetR (version 1.4.0) and ComplexUpset (version 1.3.3) were used to construct the upset plots to present the unique and shared number of ASVs and species among the different groups [[18], [19], [20]]. The MicrobiotaProcess package (version 1.6.6) was used to create rarefaction curves [21].

## Limitations

None.

## Ethics Statement

This work does not contain any experiments with humans, animals, or social media platforms.

## CRediT Author Statement

**Sreeradha Mallick:** Methodology, Investigation, Writing – review & editing; **Christina Pavloudi:** Conceptualization, Software, Data curation, Writing – review & editing; **George Joseph Chakkalakkal:** Software, Writing – review & editing; **Vladimir Lažetić:** Writing – review & editing, Funding acquisition. **Jimmy Saw:** Conceptualization, Software, Writing – review & editing. **Ioannis Eleftherianos:** Conceptualization, Supervision, Writing – original draft, Funding acquisition.

## Data Availability

ENAPRJEB85826 (Original data).

## References

[1] Mallick S., Pavloudi C., Saw J., Eleftherianos I. (2025). *Heterorhabditis bacteriophora* symbiotic and axenic nematodes modify the *Drosophila melanogaster* larval microbiome. Front. Microbiol..

[2] Ozakman Y., Eleftherianos I. (2021). Nematode infection and antinematode immunity in *Drosophila*. Trends Parasitol..

[3] Ciche T. (2007). The biology and genome of *Heterorhabditis bacteriophora*. WormBook.

[4] Ludington W.B., Ja W.W. (2020). *Drosophila* as a model for the gut microbiome. PLoS Pathog..

[5] Schissel M., Best R., Liesemeyer S., Tan Y.-D., Carlson D.J., Shaffer J.J., Avuthu N., Guda C., Carlson K.A. (2021). Effect of Nora virus infection on native gut bacterial communities of *Drosophila melanogaster*. AIMS Microbiol..

[6] Trienens M., Rohlfs M. (2020). A potential collective defense of *Drosophila* larvae against the invasion of a harmful fungus. Front. Ecol. Evol..

[7] Barron A.J., Agrawal S., Lesperance D.N.A., Doucette J., Calle S., Broderick N.A. (2024). Microbiomederived acidity protects against microbial invasion in *Drosophila*. Cell Rep..

[8] Heryanto C., Ratnappan R., O’Halloran D.M., Hawdon J.M., Eleftherianos I. (2022). Culturing and genetically manipulating entomopathogenic nematodes. J. Vis. Exp..

[9] Kenney E., Hawdon J.M., O’Halloran D., Eleftherianos I. (2019). *Heterorhabditis bacteriophora* excreted-secreted products enable infection by *Photorhabdus luminescens* through suppression of the Imd pathway. Front. Immunol..

[10] Castillo J.C., Creasy T., Kumar P., Shetty A., Shokal U., Tallon L.J., Eleftherianos I. (2015). *Drosophila* anti-nematode and antibacterial immune regulators revealed by RNA-seq. BMC Genomics.

[11] O'Cathail C., Ahamed A., Burgin J., Cummins C., Devaraj R., Gueye K. (2025). The European nucleotide archive in 2024. Nucleic Acids Res..

[12] Quast C., Pruesse E., Yilmaz P., Gerken J., Schweer T., Yarza P., Peplies J., Glöckner F.O. (2013). The SILVA ribosomal RNA gene database project: improved data processing and web-based tools. Nucleic Acids Res..

[13] McMurdie P.J., Holmes S. (2013). Phyloseq: an R package for reproducible interactive analysis and graphics of microbiome census data. PLoS One.

[14] Chao A. (1984). Non-parametric estimation of the classes in a population. Scand. J. Stat..

[15] Chao A., Lee S.-M. (1992). Estimating the number of classes via sample coverage. J. Am. Stat. Assoc..

[16] Segata N., Izard J., Waldron L., Gevers D., Miropolsky L., Garrett W.S., Huttenhower C. (2011). Metagenomic biomarker discovery and explanation. Genome Biol..

[17] Cao Y., Dong Q., Wang D., Zhang P., Liu Y., Niu C. (2022). MicrobiomeMarker: an R/bioconductor package for microbiome marker identification and visualization. Bioinformatics.

[18] Lex A., Gehlenborg N., Strobelt H., Vuillemot R., Pfister H. (2014). UpSet: visualization of intersecting sets. IEEE Trans. Vis. Comput. Graph..

[19] Nusrat S., Harbig T., Gehlenborg N. (2019). Tasks, techniques, and tools for genomic data visualization. Comput. Graph. Forum.

[20] Krassowski M., Das V., Sahu S.K., Misra B.B. (2020). State of the field in multi-omics research: from computational needs to data mining and sharing. Front. Genet..

[21] Xu S., Zhan L., Tang W., Wang Q., Dai Z., Zhou L., Feng T., Chen M., Wu T., Hu E., Yu G. (2023). MicrobiotaProcess: a comprehensive R package for deep mining microbiome. Innovation.

